# Methylation of the glucocorticoid receptor gene NR3C1: a summary for clinicians working with children and families

**DOI:** 10.1192/bjo.2021.643

**Published:** 2021-06-18

**Authors:** Victoria Brown

**Affiliations:** NHS Education for Scotland. NHS Grampian

## Abstract

**Aims:**

It has been shown that the glucocorticoid receptor NR3C1 gene can be methylated (“switched off”) in response to early adversity. Methylation has also been linked to physiological changes in the body's response to stress by changing the sensitivity of the hypothalamic-pituitary-adrenal (HPA) axis. In adults, associations have been made between NR3C1 methylation and borderline personality disorder, depression and post-traumatic stress disorder. Environmental and social co-variates increase with lifespan so establishing cause and effect is difficult. Studies in children, then, may illuminate patterns to inform current hypotheses.

This paper reviews the literature on children and adolescents linking glucocorticoid gene receptor NR3C1 to the psychopathology of mental illness. Findings are presented in an accessible manner to engage people less familiar with genetics and to inform frontline clinicians of this quickly growing area of research.

**Method:**

MEDLINE and PsychINFO were searched for relevant peer-reviewed original research using the following keywords and associated mesh terms: NRC31, glucocorticoid receptor gene, methylation, epigenetics, child, adolescent, trauma, psychopathology, gene expression.

**Result:**

14 studies were identified involving 5475 young people. Degree of NR3C1 methylation was associated with severity of early life adversity. Methylation was linked with psychopathology including borderline personality disorder, internalising symptoms and externalising symptoms with sex differences. The most consistent association was with depression. Methylation seems to modulate the interaction between environment and genetics with the suggestion that the effect may be protective in some cases. However, longitudinal genetic sampling was only conducted in one study.

**Conclusion:**

Heterogeneity of studies in the epigenetics field are discussed but should not detract from future possibilities. The hope is to identify therapeutic targets or monitor response to treatment as we work to better understand the biology of developmental psychology, mental illness and resilience. There is a growing understanding that epigenetic modifications likely change over time and clinical significance is most likely dictated by changes at multiple gene locations. Thus future research may need to move away from single gene research typically employed in favour of longitudinal whole genome studies in larger population studies.

It is time that clinicians integrate the concepts of “epigenetic adaptation to environmental stress” with “nature vs. nurture” in their psychoeducation with families. To understand that one's problems might be the symptom of environmental mismatch, and potentially reversible too, can bring validation and hope to families.

